# Free thoracodorsal artery polyfoliate perforator flaps for repairing multiple soft tissue defects in the hand

**DOI:** 10.1186/s12893-021-01359-0

**Published:** 2021-10-05

**Authors:** Wenquan Ding, Jianbo Xue, Yingling Zhou, Lingfeng He, Xiaofeng Wang

**Affiliations:** Department of Hand Surgery, Department of Plastic Reconstructive Surgery, Ningbo No. 6 Hospital, Ningbo, 315040 China

**Keywords:** Microsurgery, Hand injury, Polyfoliate flaps, Multiple wounds, Soft tissue defects, Hand function

## Abstract

**Background:**

Hand injury is commonly associated with multiple soft tissue defects. Polyfoliate flaps grafting is the optimal approach for multiple wounds.The feasibility of clinical using of free thoracodorsal artery polyfoliate perforator flaps for repairing multiple soft tissue defects in the hand needs to be confirmed in clinical practice.

**Methods:**

Fifteen patients with hand soft tissue defects that were repaired using free thoracodorsal artery polyfoliate perforator flaps from January 2015 to February 2018 was retrospectively analysed. The survival rate, the operative time, the appearance and sensory recovery of the flaps, and hand function were evaluated.

**Results:**

The flaps of all 15 patients survived. Vascular crisis occurred in one patient, and the flap was saved after exploratory operation. The 15 patients were followed up for 12–26 months. Sensation in the flaps was partially recovered in all 15 patients. The wound in the donor area was closed directly with sutures. Mean score of scars at the donor site were assessed using the modified Vancouver scar scale (VSS) was 2.7. A puffed appearance in the recipient area was noted in four patients. To obtain a more satisfactory appearance, revision of the flap was performed once in these four patients. The Total Active Movement (TAM) evaluation system was used to assess the results, which were considered excellent in seven patients, good in six patients, fair in two patients, and poor in none of the patients. Ten of the 15 patients returned to their primary jobs.

**Conclusion:**

Free thoracodorsal artery polyfoliate perforator flaps are appropriate for repairing multiple soft tissue defects in the hand, offer a satisfactory appearance, require a short operative time, and have little impact on the function and aesthetics of the donor site.

## Background

Hand injury is usually associated with multiple wounds with deep tissue (bone or tendon) exposure [1, 2]. For the repair of multiple isolated wounds, multiple flaps (two or more pedicles) are not an option because the recipient area has limited blood vessels for anastomosis. The potential options include (1) the use of a large flap to cover one enlarged wound formed by the expansion of multiple isolated wounds in stage I surgery, followed by revision of the flap in stage II surgery [3], or (2) repair of the wounds with polyfoliate flaps in single-stage surgery [4, 5]. Option 1 is a relatively easy approach; however, functional exercises cannot be carried out early due to the limitation of the intact flap, and hand movement may be restricted. A polyfoliate flap is an ingenious solution for covering multiple isolated wounds. However, designing and harvesting polyfoliate flaps are challenging because the flaps are small, and each flap needs an independent perforator [5].

To date, the most commonly used polyfoliate flaps are anterolateral thigh polyfoliate perforator flaps [4, 6]. Anterolateral thigh polyfoliate perforator flaps are an improved version of anterolateral thigh perforator flaps, and the technique used to harvest these flaps is well established. However, these flaps have the following shortcomings: (1) the flaps are thick, especially when taken from obese patients. The flaps are not ideal for the repair of wounds in areas covered by thin skin, such as the fingers; (2) the presence of multiple perforator branches in the descending branch of the lateral circumflex femoral artery is a prerequisite for creating polyfoliate flaps. Multiple perforator branches are usually thinner vessels from a deep origin vessel. The pathways of the perforators from the origin vessel to the skin are long. Intramuscular dissection is tedious and challenging, and the perforators are prone to damage; and (3) the donor site is not adequately concealed, and the scars at the donor site will be visible when the patient wears shorts.

The thoracodorsal artery perforator flap has great potential for the repair of large and relatively superficial wounds in the upper extremity due to its suitable skin colour, texture, and thickness as well as its hidden donor site [7], reliable vascular pedicle [8], and ability to combine with muscle tissue to form a chimeric flap [9, 10]. With recent research trends focusing on reducing damage and local scarring at the donor site and expanding the clinical application of thoracodorsal artery perforator flaps, we conducted a study on the use of free thoracodorsal artery polyfoliate perforator flaps to repair soft tissue defects wounds in the hand.

The purposes of our study was to confirm the feasibility of using free thoracodorsal artery polyfoliate perforator flaps for repairing multiple soft tissue defects in the hand, observe the appearance of the donor area and the recovery of hand function after surgery, and explore the advantages and disadvantages of this technique.

## Methods

This study was approved by the ethics committee of Ningbo No. 6 Hospital. All patients in this study were informed of the surgical plan and follow-up examinations in writing before surgery and signed an informed consent form.

### Clinical application of free thoracodorsal artery polyfoliate perforator flaps

#### Surgical technique

A Siemens 64-slice dual-source CT and colour Doppler ultrasound diagnostic apparatus (Siemens Acuson Sequoia 512, Germany) was used to scan and locate the perforators of the thoracodorsal arteries in the donor areas of the patients. Major and long perforators were selected, and their courses, the locations of the exit points, the approximate diameters of the perforators, and adjacent structures were marked.

The procedure was performed under general anaesthesia. Briefly, the patient was placed in the lateral decubitus position with abduction of the shoulder on the donor area side. The operative field was disinfected.

The anterior edge of the latissimus dorsi muscle and the scapula were marked. The connecting line between the posterior axillary wall and the posterior superior iliac spine was used as a reference axis to design the flap. According to the sizes and shapes of the hand wound defects, we designed the flap along the major thoracodorsal artery perforators. The area size of flap was appropriately enlarged according to the thickness of the fat at the donor site.

According to the design line of the flap, an incision was made at the medial edge of the flap. Separation was performed medially from the subcutaneous tissue layer towards the lateral side until the marked points of the major perforators were reached. Then an incision was made along the design line to incise the lateral edge of the flap. While vascular pedicles of adequate length were completely developed, the flap was divided to polyfoliate flaps with the pedicle intact (Figs. 2A, 4A). Thus, each flap had an independent perforator. The wound at the donor site was directly closed with sutures.

Then, the patient was placed in the supine position. The vessels of the flap were perfused using diluted heparin and lidocaine to identify open branches from the pedicle. Any open branches of each perforator were ligated. Then, the flap was moved to the recipient area and secured with intermittent sutures. The artery of the pedicle was anastomosed to an artery in the recipient area. An accompanying vein of the pedicle was anastomosed to an accompanying vein of the artery in the recipient area, and the other accompanying vein was anastomosed to the superficial vein. End-to-end anastomosis was used in this procedure unless a large diameter difference between the two vessels was evident. If a large difference was evident, end-to-side anastomosis was used (Figs. 2B, 4B).

After surgery, the patient was admitted to the flap transplantation intensive care unit where he or she was treated with anti-inflammatory, antispasmodic, and anticoagulant agents. The patient was cared for in a quiet room with a temperature of 25–28 °C and maintained complete bed rest for 1 week. Assigned staff observed the blood supply of the flaps every hour.

#### Surgical subjects

Patients with hand skin and soft tissue defects who were admitted to Ningbo No. 6 Hospital (Ningbo, China) from January 2015 to February 2018 and met the inclusion criteria were included in the study. The inclusion criteria were: (1) multiple skin and soft tissue defects in the hand due to recent trauma, (2) deep tissues exposed, such as bone and tendon, and (3) lack of availability of a single flap to repair all the wounds. The exclusion criteria were: (1) patient’s age > 60 years, (2) patient who can’t cooperate with complete bed rest, (3) severe arteriosclerosis in the blood vessel of the recipient area, (4) wound infection, (5) the area of skin defect exceeds the coverage of thoracodorsal artery perforator flap, and (6) restrictions to free flap use due to damaged blood vessels in the recipient area. After being informed of the details of the surgery, the patients chose the surgical plan and signed the informed consent form for surgery and for participation in this study.

Fifteen patients were enrolled in the study, including 12 men and three women, with an average age of 37.7 years (range: 19 to 48 years). The causes of injury included crush injury (11 patients), thermal pressure injury (three patients), and car accident injury (one patient). The injury sites included the fingers (six patients), the dorsum of hand (six patients), and the dorsum of hand combined with the fingers (three patients). All patients presented with bone or extensor tendon exposure, and three patients had extensor tendon defects. The mean skin defect area (cm^2^) was 25.2 (range: 15 to 43). Wound debridement was performed in all 15 patients during emergency surgery, and the wounds were covered with flaps approximately 1 week later once the wounds were free of infection.

Case 1: Free thoracodorsal artery polyfoliate perforator flaps for the repair of skin defects on the dorsum of the right hand and the dorsal side of the thumb.

**Fig. 1:**
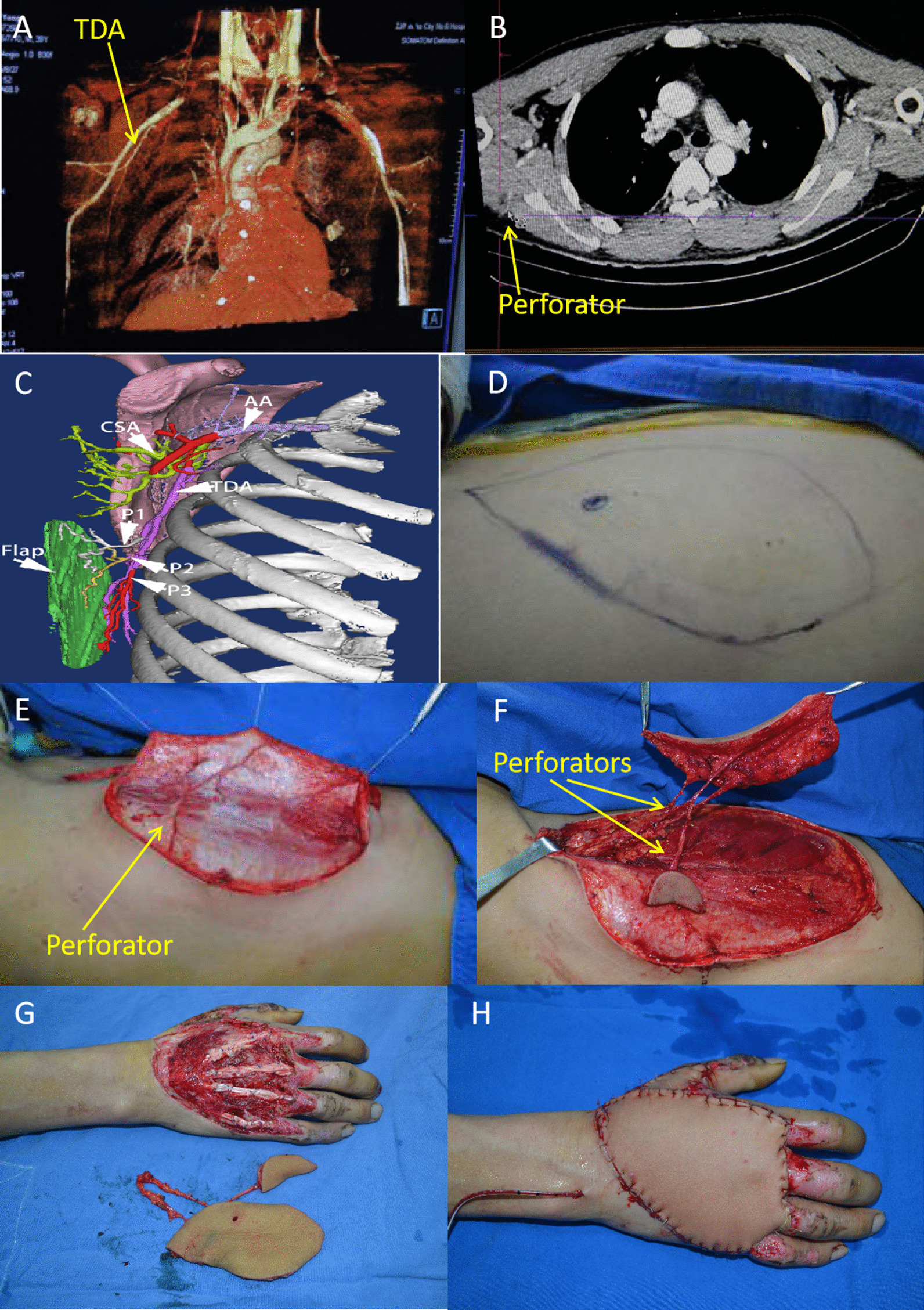
A 40-year-old man with skin defects on the dorsal side of his right hand and thumb combined with defects of the thumb extensor tendon caused by a crush injury underwent wound repair with free thoracodorsal artery polyfoliate perforator flaps and tendon repair. **A** CTA image showing the thoracodorsal artery; **B** Two-dimensional CTA image showing the thoracodorsal artery; **C** Flap design simulation. (*AA* axillary artery, *CSA* circumflex scapular artery, *TDA* thoracodorsal artery, *P1*–*P3* perforators); **D** The flap design was based on the perforator location detected by CTA and ultrasound; **E** Dissection of the flap showed perforators entering the flap; **F** The separated flap and multiple perforators entering the flap. The flap was modified to polyfoliate flaps; **G** After grafting repair of the tendon, the flaps were used to cover the wound in the hand; **H** The appearance immediately after covering

**Fig. 2:**
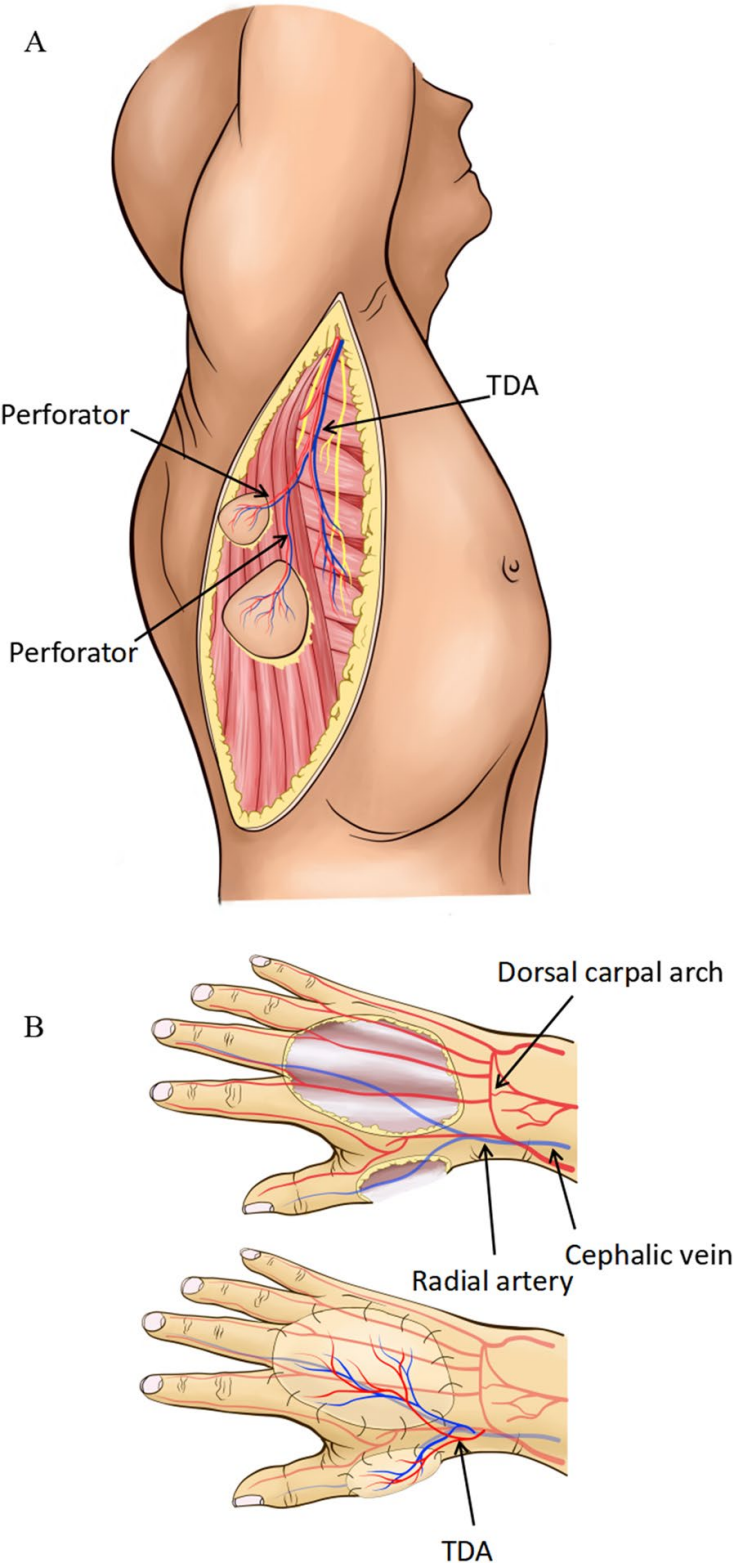
The operation diagram of Case 1. **A** Diagram of flap donor site; **B** Diagram of recipient site

A 40-year-old man presented with a crush injury of the right hand and skin defects on the dorsal side of the hand and thumb. CTA and colour Doppler ultrasound were performed to locate the perforators, and then free thoracodorsal artery polyfoliate perforator flaps were used to repair the defects. The artery and veins of the pedicle were anastomosed (end-to-end) to the radial artery, the radial vein, and the cephalic vein, respectively. The flap survived with a good blood supply. The wound at the donor site was closed directly (Figs. 1, 2).

Case 2: Free thoracodorsal artery polyfoliate perforator flaps for the repair of skin defects on the right index and middle fingers.

**Fig. 3:**
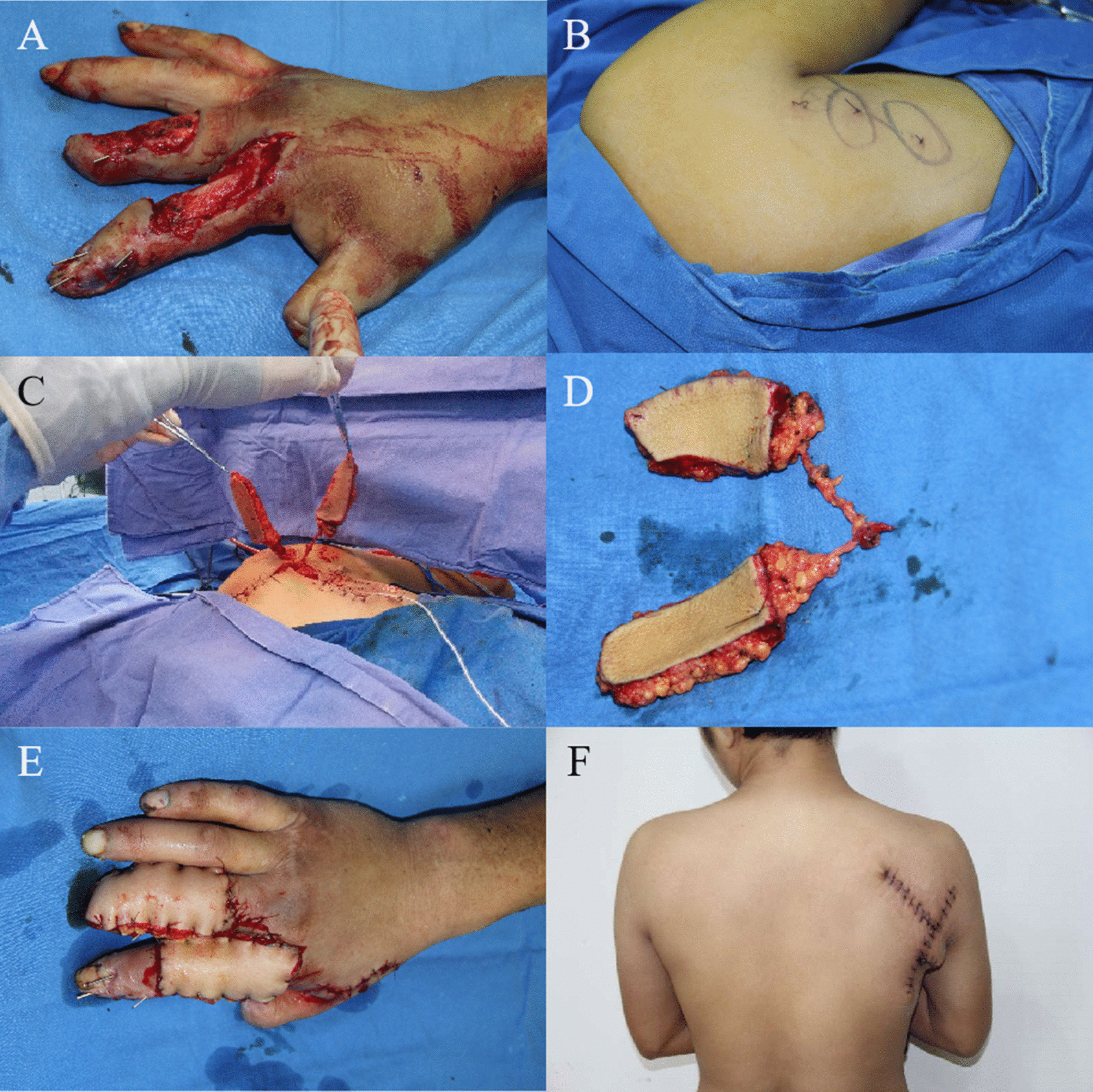
A 23-year-old man with wound defects on the back of his right index and middle fingers underwent wound repair using free thoracodorsal artery polyfoliate perforator flaps. **A** Wound defect on the dorsum of the right index and middle fingers; **B** Design of the thoracodorsal artery perforator flap; **C**, **D** The flaps separated; **E** Coverage of the wound by the free thoracodorsal artery polyfoliate perforator flaps; **F** Direct closure of the wound at the donor site with relatively invisible scars

**Fig. 4:**
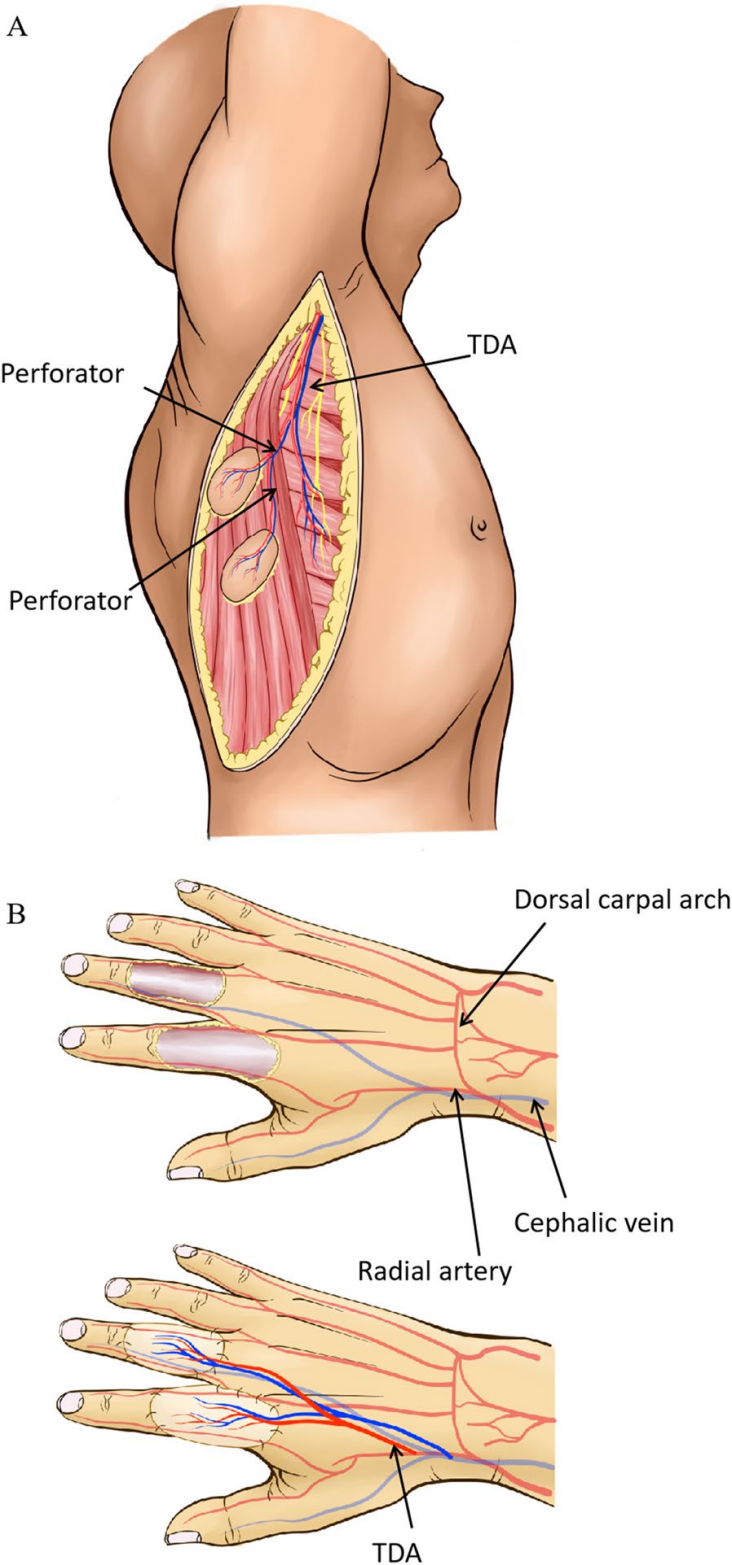
The operation diagram of Case 2. **A** Diagram of flap donor site; **B** Diagram of recipient site

A 23-year-old man was admitted to the hospital due to soft tissue defects in the right index and middle finger caused by a car accident. After initial debridement, free thoracodorsal artery polyfoliate perforator flaps were used to repair the wound. The artery and veins of the flap were anastomosed (end-to-end) to the radial artery, radial vein, and the cephalic vein, respectively. The flap survived. The wound at the donor site was directly closed with sutures and was relatively invisible (Figs. 3, 4).

### Follow-up and assessment

For these 15 patients, the flap survival rate, reoperation rate, and operative time were documented. The patients were followed up regularly after the operation. The patients were evaluated periodically during the follow-up using subjective and objective parameters. The evaluation included the degree of sensory recovery of the flap, hand function recovery, a puffed appearance of the flap at the recipient site, single or multiple revisions of the flap, readiness to return to work. The MRC scale for sensory recovery modified by Mackinnon and Dellon [11] (S0, S1, S2, S3, S3+, and S4 correspond to scores of 0–5, respectively) was used to assess the sensation function of the flap. Scars at the donor site were assessed using the Vancouver scar scale (VSS) modified by Moon [12], which rates scars according to the sum of four subscales (pigmentation, vascularity, pliability, and height). Hand function was evaluated using the Total Active Movement (TAM) system recommended by the American Society of Hand Surgery in 1975.

## Results

### Surgery and functional recovery of the patients

The surgical findings and follow-up results for the patients are shown in Table 1. The operative time was 138.1 (120–175) min. All 15 flaps survived. In one patient, vascular crisis occurred 2 days postoperatively. Exploratory surgery showed that the crisis was caused by a haematoma compressing the vein. After the haematoma was removed, the vascular crisis was resolved.

**Table 1:** Postoperative follow-up evaluation form (latest follow-up)

Follow-up parameters	Results
Time between injury and surgery (days), mean (range)	7.1 (5–12)
Number of surviving flaps	15/15
Number of patients who underwent reoperation for flap exploration	1/15
Operative time (minutes), mean (range)	138.1 (120–175)
Follow-up period (months), mean (range)	17.8 (12–26)
Sensory scale score, mean (range)	1.7 (1–3)
Vancouver scar scale (VSS) modified by Moon, mean (range)	2.7(1–5)
Number of patients with a puffed appearance of flaps in the recipient area	4/15
Number of patients who underwent flap revision once	4/15
Hand function score: Excellent	7
Good	6
Fair	2
Poor	0
Number of patients who returned to their primary jobs	10/15

**Fig. 5:**
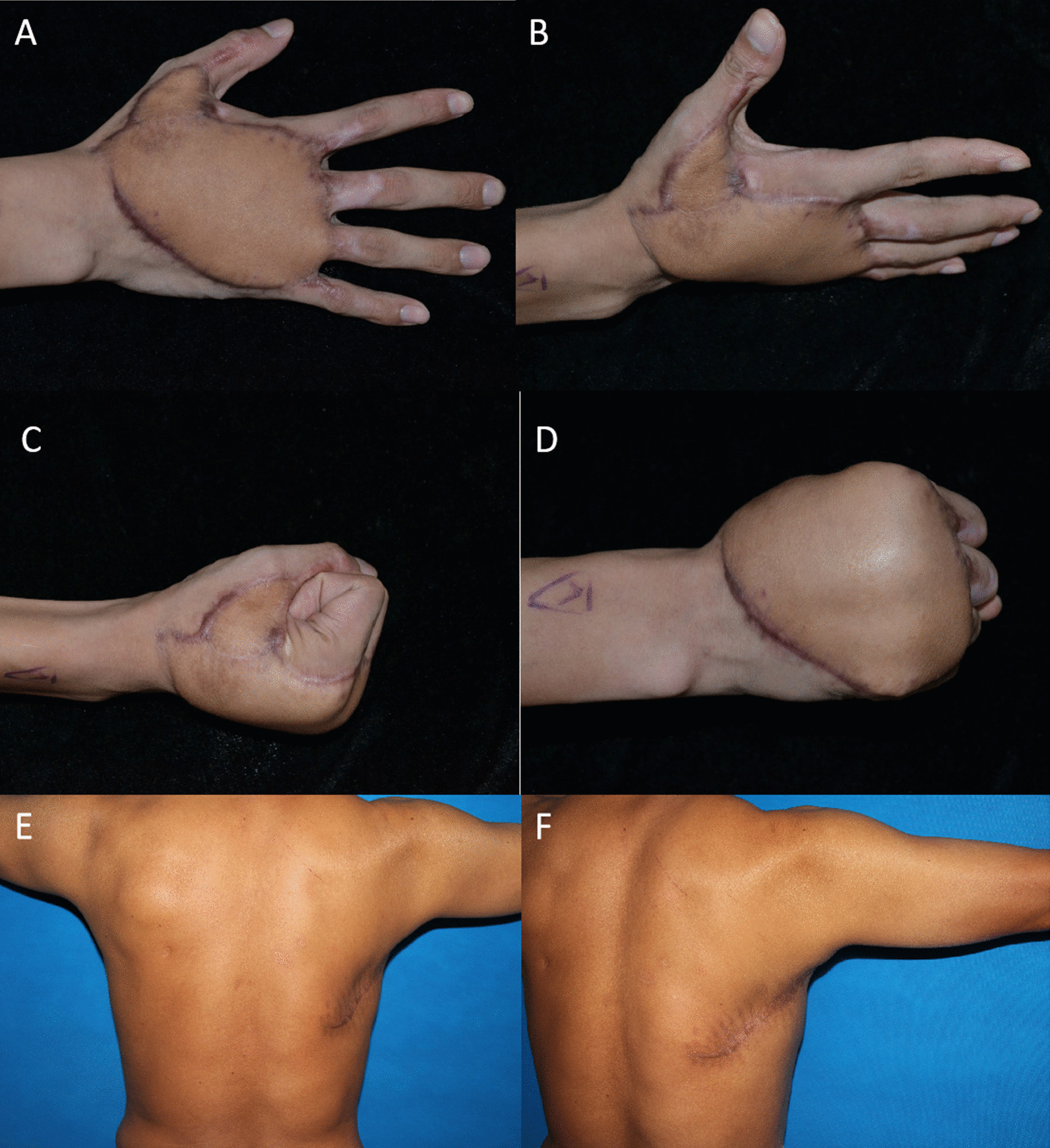
Case 1’s follow-up 12 months after surgery. **A**, **B** The flap healed well, without an obvious puffed appearance; **C**, **D** Good recovery of flexion and extension functions in the fingers; **E**, **F** Scars at the donor site are relatively invisible

For Case 1, a follow-up examination 12 months after surgery showed that the flaps had healed well, without a puffed appearance. No flap revision was performed. The flexion and extension functions of the fingers recovered well. The scars at the donor site were relatively invisible. Sensation in the flap was partially recovered. The patient returned to his primary job (Fig. 5).

**Fig. 6:**
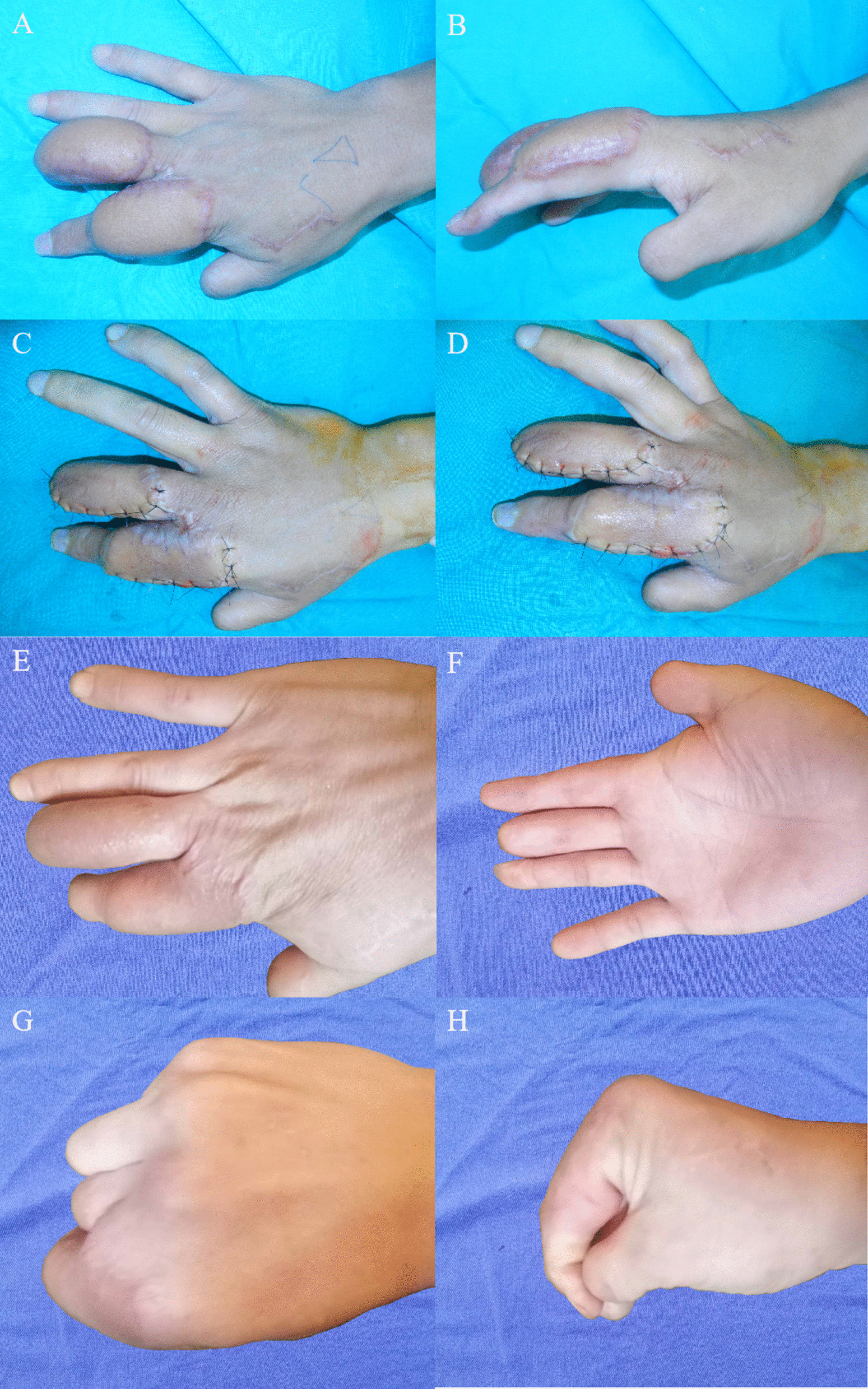
Case 2’s 6-month follow-up after surgery. **A**, **B** A slightly puffed appearance of the flap; **C**, **D** Satisfactory appearance after flap revision; **E**, **F** A well-healed flap without a puffed appearance at the 24-month follow-up visit after surgery; **G**, **H** Good recovery of flexion and extension functions in the index and middle fingers

In Case 2, a puffed appearance of the flap was noted 6 months after surgery. Thus, flap revision was performed once, and a satisfactory appearance was obtained. After 24 months of follow-up, the flaps had healed well, and no obvious puffed appearance of the flap was noted. The index and middle fingers showed good recovery of flexion and extension functions. Sensation in the flap was partially recovered. The patient returned to his primary job (Fig. 6).

### Follow-up and evaluation

The average follow-up time was 17.8 months (12–26 months). In terms of sensory recovery, sensation in the flaps was partially recovered in all 15 patients, but the degree of sensory recovery was low. In this study, the wound at the flap donor area was directly sutured, and mean score of scars at the donor site were assessed using the modified Vancouver scar scale (VSS) was 2.7. No adverse impact on the range of motion of the shoulder on the affected side was reported. The position and appearance of the breast in female patients were not affected. A puffed appearance of the flaps in the recipient area was reported for four of the 15 patients. To obtain a more satisfactory appearance, revision of the flap was performed once in these four patients. The TAM evaluation system was used to assess the movement outcomes, which were considered excellent in seven patients, good in six patients, fair in two patients, and poor in none of the patients. Ten of the 15 patients returned to their primary jobs (Table 1).

## Discussion

According to this study, the feasibility of using free thoracodorsal artery polyfoliate perforator flaps for repairing multiple soft tissue defects in the hand was confirmed. During the operation, we found that thoracodorsal artery contained several constant perforating branches, which can be used to design polyfoliate flaps. The flaps are sufficient for concurrent repair of multiple wounds or tissue defects. This is consistent with findings of Angrigiani in the cadaver study [13]. In this studies, we found that the thoracodorsal arteries originate from the subscapular artery and travel along the deep surface of the latissimus dorsi muscle, with medial and lateral branches at a site approximate 8 cm below the posterior axillary wall and 2 cm behind the anterior edge of the belly of the latissimus dorsi muscle. The lateral branch is larger and has two accompanying veins. The diameter of the largest perforator was about 0.4–0.6 mm. Polyfoliate flaps can be designed in this area.

In this study, the operative time in this study was 138.1 (120–175) min, which was significantly shorter than the time required for free anterolateral thigh flap grafting (usually > 3 h) [14, 15]. In thoracodorsal artery polyfoliate perforator flaps, perforators travel superficially in the intermuscular space and are easier to separate than those in the anterolateral thigh flap. The courses of the perforators are relatively constant. Their diameters are relatively large, and the pedicle is easy to anastomose. Therefore, the operative time is shorter. The vessels can be harvested extending to the main trunk of the thoracodorsal artery and anastomosed with the larger vessels in the recipient area, so the flap survival rate is increased.

During the operation and postoperative follow-up, we found that the donor site is adequately concealed, and the wound at the donor site can be closed with direct suturing if the width of the wound is less than 9 cm. In this study, mean score of scars at the donor site were assessed using the modified Vancouver scar scale (VSS) was 2.7. The healed donor site has an inconspicuous and covert scar and limited loss of function [10, 16]. Patients with a wound width of 9 cm or more will not be selected such that the wound at the donor site cannot be directly closed with low morbidity.

The disadvantages of free thoracodorsal artery polyfoliate perforator flaps include the following: (1) vascular spasm may occur during dissection and separation. Therefore, microsurgical experience was needed; (2) during the operation of flap separation and transplantation, the patient’s position needs to change.

The limitation of this study was that sensation in the flap recovered only slightly. Because no sensory nerves were repaired in the flaps in this group of patients, after the wounds were covered with the flaps, sensation recovery maybe relied on the small nerves around the wounds regenerating into the flap and providing innervation. Therefore, the degree of flap sensation is generally relatively low. If this type of flap is used to repair wounds on the palm side of the fingers or hands, the use of a flap with a cutaneous nerve that can be anastomosed to the digital nerve when the flap covers the wound is recommended, which will effectively improve the sensory recovery of the flap. There is a challenge that, in the polyfoliate perforator flaps, each flap can not be guaranteed containing a long enough cutaneous nerve, so it can not be guaranteed that each flap will have innervation in the future. In further study, autologous or allogeneic nerve graft combined with nerve end-to-side anastomosis could be studied as a solution.

## Conclusion

Free thoracodorsal artery polyfoliate perforator flaps are appropriate for repairing multiple soft tissue defects in the hand, offer a satisfactory appearance, require a short operative time, and have little impact on the function and aesthetics of the donor site.

## Data Availability

All data generated or analyzed during this study are included in this published article.
